# Case Report: Autosomal dominant polycystic kidney disease and Wilms’ tumor in infancy and childhood

**DOI:** 10.3389/fped.2024.1322142

**Published:** 2024-03-21

**Authors:** Doviltyte Zina, Kiudeliene Rosita, Zviniene Kristina, Rutkauskiene Giedre, Masalskiene Jurate

**Affiliations:** ^1^ Medical Academy, Lithuanian University of Health Sciences, Kaunas, Lithuania; ^2^ Department of Pediatrics, Medical Academy, Lithuanian University of Health Sciences Kaunas Clinics, Kaunas, Lithuania; ^3^ Department of Radiology, Medical Academy, Lithuanian University of Health Sciences Kaunas Clinics, Kaunas, Lithuania

**Keywords:** Wilms tumor, polycystic kidneys, ADPKD, *PKD1* gene, nephroblastoma, nephrobastomatosis

## Abstract

**Background:**

Autosomal dominant polycystic kidney disease (ADPKD) is rare but one of the most common inherited kidney diseases. Normal kidney function is maintained until adulthood in most patients. About 7 in 10 patients with ADPKD develop kidney failure in the latter half of their fifth decade of life. Wilms' tumor, or nephroblastoma, is the most common malignant tumor stemming from kidney cells in the pediatric age group. This type of tumor is the most frequently occurring kidney malignancy in children between the ages of 0 and 5 years. The exact cause of Wilms' tumor is unknown, though about 10% of cases have a genetic predisposition. Wilms' tumor is one of the most successfully treated childhood oncological diseases. Overall, the 5-year survival rates were approximately 90% in both the National Wilms Tumor Study (NWTS) and Paediatric Oncology SIOP studies, showing similar results.

**Case presentation:**

We report a case of a girl diagnosed with autosomal polycystic kidney disease, who subsequently developed Wilms' tumor and underwent successful treatment with chemotherapy. Polycystic kidney disease was suspected in the fetus during prenatal ultrasound and confirmed after birth with ultrasound and genetic testing. The Wilms tumor was an accidental finding during abdominal MRI at the age of 2 years old to rule out liver pathology.

**Conclusion:**

Reports on whether a diagnosis of ADPKD is a risk factor for malignancy are conflicting. In this particular case, Wilms' tumor is present in the background of polycystic kidney disease and was timely diagnosed by an incidental MRI.

## Introduction

Autosomal dominant polycystic kidney disease (ADPKD) is one of the most common progressive kidney diseases. It ranks as the fourth most common cause for kidney replacement therapy worldwide (1). The incidence of this disease is estimated to be about 1 in 400 to 1 in 1,000 people (2). Approximately 70% of patients with ADPKD progress to end-stage kidney disease (ESRD) in the latter half of their fifth decade of life (3). In most cases, ADPKD is caused by pathogenic variants in *PKD1* (chromosome *16p13.3*), which encodes polycystin-1, and *PKD2* (chromosome *4q21*), which encodes polycystin-2 (2). About 9% of cases result from de novo pathogenic variants (4). *PKD1* is usually associated with a more severe course of the disease than *PKD2*. It is diagnosed at an earlier age, presents with more kidney cysts, earlier onset of arterial hypertension, and faster progression to ESRD compared to *PKD2* (4). Cyst development begins at an early age, and macroscopic cysts can become detectable in childhood (5).

Wilms’ tumor (WT), also known as nephroblastoma, is a malignant embryonal tumor that develops due to impaired kidney organogenesis, characterized by the proliferation of metanephric blastema cells (6). WT is the most common type of kidney tumor in children, accounting for approximately 80% of kidney tumors in children and teenagers. It is relatively rare, comprising about 5% of all malignant tumors in childhood. Around 15 syndromes are associated with WT. Only 10% of WTs are associated with pathogenic genetic variants; in most cases, the etiology of the disease remains unknown (7). It occurs equally frequently in both genders and is typically detected in children under the age of 5 years (8). The main characteristic of this illness is the presence of a tumor, often asymptomatic, detected incidentally by noticing or palpating a larger abdominal volume. However, about 35% of cases exhibit clinical signs such as hypertension, hematuria, and flank pain. In rare cases, the tumor can rupture and cause bleeding into the surrounding tissue (7, 8). Nephroblastomatosis is a rare premalignant condition associated with WT, presenting as multiple foci derived from abnormal nephrogenic cells. This condition is often asymptomatic, which leads to a late diagnosis of advanced WT and delayed treatment with chemotherapy. MRI is the best test to be used in diagnosing nephroblastomatosis (9, 10). According to the literature, Wilms' tumors present very rarely in childhood in the background of ADPKD.

## Case report

A female newborn was delivered at 34 weeks of gestation, from the second pregnancy and the second delivery. Polycystic kidney disease was suspected in the fetus during prenatal ultrasound. At birth, her weight was 2,680 g (90th percentile), and her length was 47 cm (50th percentile). On physical examination, she exhibited small, low-set ears, a protruding forehead, hypertelorism, epicanthus, a sunken bridge of the nose, and clinodactyly of the second finger. Both her mother and grandmother had been diagnosed with polycystic kidney disease.

After birth, a kidney ultrasound revealed increased kidney echogenicity and multiple cysts measuring up to 0.3 cm in diameter. Arterial hypertension was diagnosed at the age of 2 months, and treatment with angiotensin-converting enzyme inhibitors (ACEi) was initiated. Additionally, at the age of 2 months, the girl was diagnosed with a urinary tract infection caused by *Escherichia coli*. At 4 months of age, compensated metabolic acidosis was diagnosed, and treatment with sodium bicarbonate proved effective.

**Figure 1:**
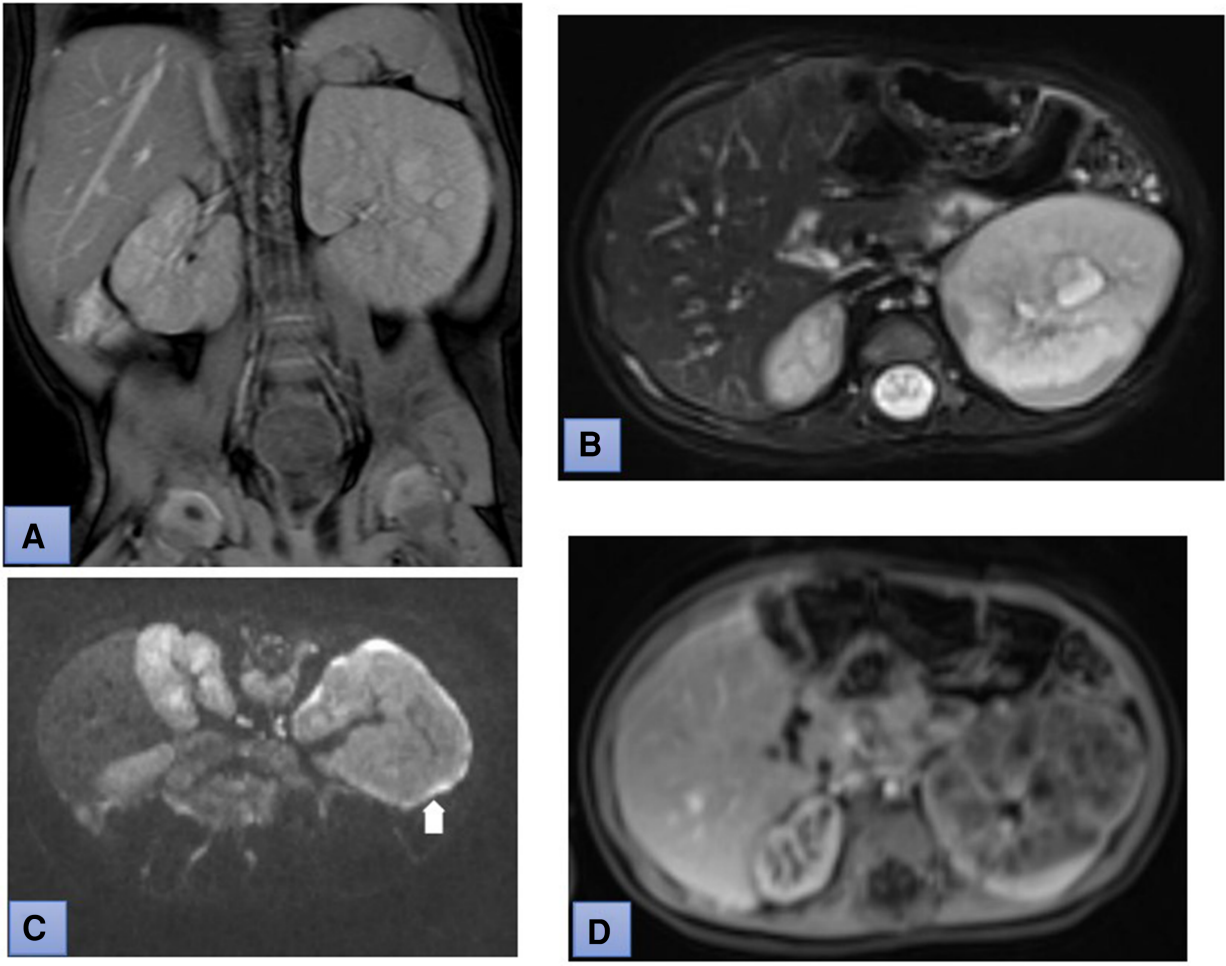
Coronal T2-weighted (**A**) and fat suppressed T2-weighted (**B**) MR images demonstrate enlarged both kidneys. Diffusion-weighted MRI (DWI) (*b*-value: 800) and apparent diffusion coefficient (ADC) mapping show uneven thickening of the kidney capsule (white arrow in picture **C**). Postcontrast T1-weighted MR image demonstrates heterogeneous enhancement of the parenchyma of the left kidney (**D**).

**Figure 2:**
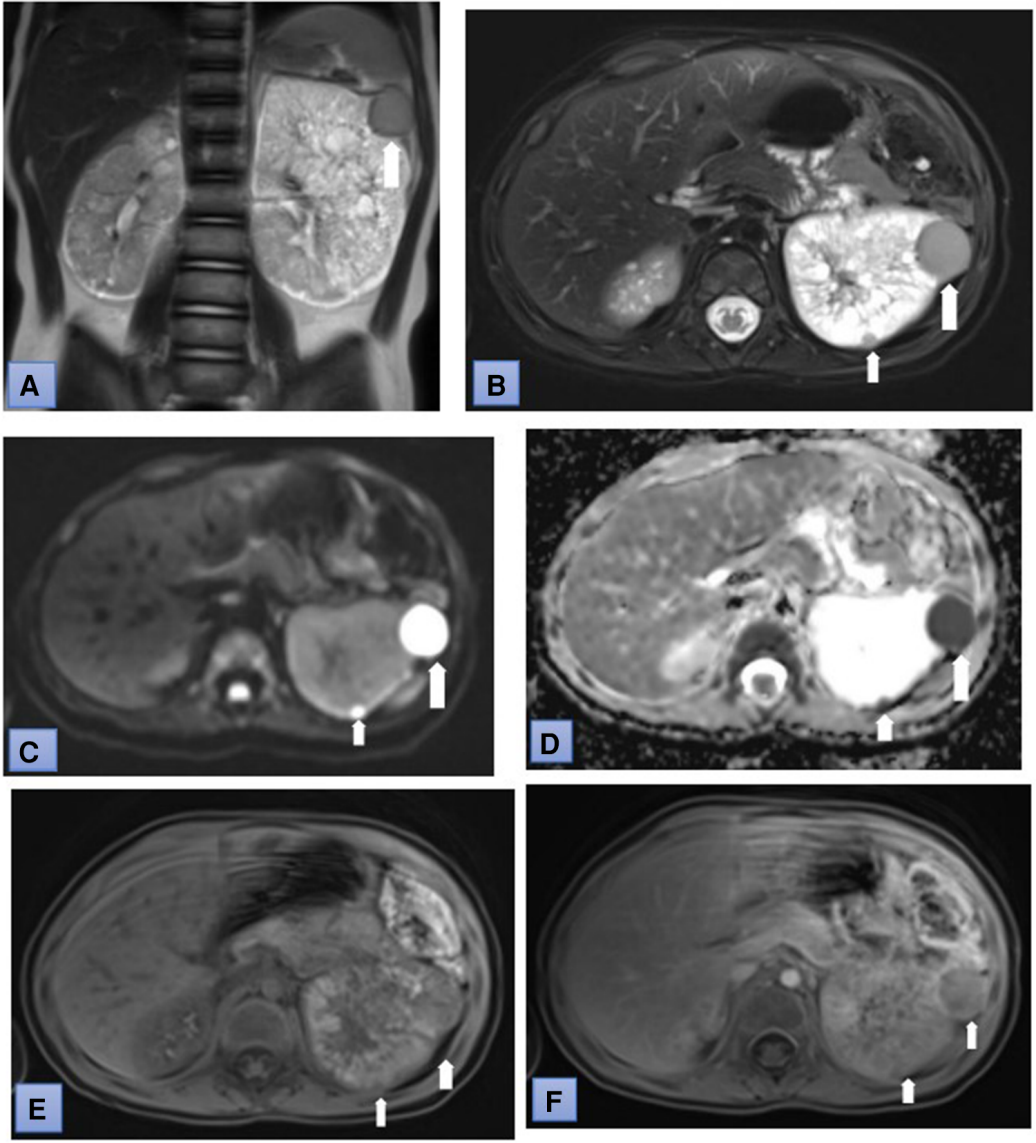
Coronal T2-weighted (**A**) and fat suppressed T2-weighted (B) MR images show several lesion in the left kidney. The lesion have restricted diffusion (**C**) and low ADC values (0.6 × 10^-3^ mm^2^/sec). (**D**) Postcontrast T1-weighted MR image: tumor demonstrates heterogenous enhancement (white arrows) during corticomedullary phase (**E**) and wash-out in nephrographic phase (**F**) (white arrows).

At the age of 1 year during follow-up, a kidney and abdominal ultrasound examination was performed. The liver parenchyma appeared diffusely heterogeneous, with formations of lesser echogenicity, measuring 0.5–1.0 cm in size, resembling observed nodules. Enlargement of both kidneys was noted: the right kidney measured 7.7 × 3.7 cm, and the left kidney measured 9.5 × 4.7 cm, respectively (the norm at the age of 1 year is about 4, 6-5, 6 × 2, 1-2, 9 cm). Multiple cysts were found in both kidneys, ranging from small to 1.6 cm; more cysts were observed in the left kidney. To clarify the diagnosis, it was decided to perform magnetic resonance imaging (MRI) of the abdomen, which confirmed the enlargement of both kidneys. The parenchyma of the right kidney appeared uneven with numerous small cysts measuring up to 1.2 cm in size. The kidney capsule was highlighted. The left kidney exhibited a non-structural upper part, hypertrophy, undifferentiated parenchymal structure, and an uneven thickening of about 0.6 cm width around it. Multiple foci up to 0.7 cm in size were visible in the liver parenchyma, resembling arteriovenous malformations (Figure 1). A genetic test was ordered to confirm the diagnosis, and a repeat MRI was scheduled after 1 year. Genetic studies revealed a normal karyotype of 46, XX. The whole exome study was conducted using the next-generation sequencing method. Complete sequence analysis of the *PKD1* gene showed a heterozygous genotype of a familial, maternally inherited pathogenic variant in intron 14 of the *PKD1* gene, causing autosomal dominant type 1 polycystic kidney disease. After one year, when the girl turned 2 years old, an MRI was repeated. The multiple foci in the liver parenchyma had disappeared. The liver lesions were suspected to be arteriovenous malformations due to hypertension. Liver changes disappeared after hypertension was corrected. The size of the right kidney measured 8.3 × 4.2 cm, and a new formation of 0.7 × 0.3 cm with signs of restriction appeared in the middle third of the anterior surface. The size of the left kidney was 9.9 × 6.4 cm. A thickening of 0.6 cm turned into a 2.0 cm × 1.9 cm tumour in the upper third of the of the lateral surface subcapsular. In the lower third, foci measuring 0.9 × 0.3 cm 0.8 × 0.2 cm and 0.6 × 0.2 cm with signs of restriction and intense accumulation of contrast material were observed. The foci in both kidneys were determined to be nephroblastomatosis based on radiological imaging. These formations were not visible during ultrasound examination (Figure 2). The Multidisciplinary team meeting decided to perform a puncture biopsy of the neoplastic formation in the left kidney because the origin of the mass was unclear. The biopsy's conclusion was a blastemal type of Wilms' tumor. Tumor cells reacted with immunolabel vimentin WT1. Proliferation index Ki67 was about 70%. No metastatic lesions were found in other organs, and kidney function was normal. Chemotherapy was initiated following the Umbrella-nephroblastoma SIOP 2016 treatment protocol for Wilms' tumor with nephroblastomatosis. After the first preoperative chemotherapy with vincristine and actinomycin, which lasted 6 weeks, a positive response to treatment was observed. The MRI showed favorable changes. WT in the left kidney had decreased from 2 to 0.9 cm and nephroblastomatosis in both kidney disappeared. After achieving a partial response to treatment, a second initial round of chemotherapy was repeated. An MRI was performed again after the second preoperative round of chemotherapy, also lasting 6 weeks, with vincristine and actinomycin. On imaging, the kidneys remained non-structural with multiple small cysts up to 1.2 cm in size. Wilms' tumor in the left kidney had disappeared. Considering that the MRI tumor formations disappeared after preoperative chemotherapy and the patient had chronic kidney disease threatening kidney failure, the multidisciplinary team meeting decided that surgical treatment was not indicated. During the multidisciplinary team meeting discussion, it was decided to follow the treatment according to the Umbrella protocol AV2 with vincristine and actinomycin for 6 months, followed by the treatment of nephroblastomatosis for 1 year with vincristine and actinomycin every 28 days. Serum biochemistry, including kidney and liver functions, electrolytes, calcium, and phosphorus, remained normal during treatment. At the end of the treatment, the girl was diagnosed with acute pyelonephritis caused by *Proteus mirabilis*.

Now the girl is 6 years old. Antihypertensive treatment with the maximum dose of ACE inhibitors was not sufficiently effective, as arterial blood pressure remained in the 95–99th percentile. Therefore, beta-blocker therapy was added. Sodium bicarbonate has been prescribed to correct metabolic acidosis. The mild intellectual disability has been diagnosed. On MRI, the kidneys remain non-structural, similar in size, with multiple small cysts up to 1.2 cm in diameter, with more cysts observed in the left kidney. The size of the cysts in the kidneys has not progressed. Hematuria and proteinuria are absent in the urine test. Kidney function remains stable, with creatinine at 36 μmol/L and urea at 6.5 mmol/L, but hyperfiltration is observed. The glomerular filtration rate, obtained using the Schwartz formula, is eGFR 159 ml/min/1.73 m^2^.

## Discussion

This case report describes an extremely rare clinical case of autosomal dominant polycystic kidney disease (ADPKD) and Wilms tumor in childhood. Wilms tumor is a rare disease, yet it is the most common kidney malignancy in children under the age of 5 years, accounting for 6%–7% of malignancies in this age group (7). Case reports of malignancies in children with a history of ADPKD are scarce. The incidence of malignancy in children with polycystic kidney disease (PKD) is currently unknown (11). Friend et al., in their clinical report, described five patients diagnosed with PKD who developed malignancies at a young age. Among them, four had a history of ADPKD: two were siblings diagnosed with familial testicular germ cell tumors, while the other two were diagnosed with kidney rhabdoid tumors and perivascular epithelioid cell tumors with liver metastases. One patient with autosomal recessive polycystic kidney disease was diagnosed with hepatocellular carcinoma with pulmonary metastases. Treatment applied to all patients was successful, and they all remain alive (11). The data from this study suggests an estimated incidence of 260 cases of cancer per 100,000 children with PKD per year. This estimate is likely higher than expected, considering that ADPKD is often asymptomatic during childhood. Therefore, it is possible that some children may have succumbed to malignancy without a known genetic diagnosis of PKD (11). Thankamony et al., in a clinical case report, described a child with bilateral PKD and PHACE syndrome. This syndrome encompasses posterior fossa malformation, large hemangioma, anatomical anomalies of the cerebral or cervical arteries, cardiac, and eye abnormalities. Wilms tumor developed in the right polycystic kidney at the age of 17 months. Treatment was successfully managed with kidney parenchyma-preserving partial nephrectomy, and combination chemotherapy was used (12). Gucev et al. described an 8.5 year old girl with WAGR syndrome and bilateral PKD. WAGR syndrome includes WT, genitourinary anomalies, aniridia, and intellectual disability. WT developed in the right kidney, and the child was treated with nephrectomy followed by chemotherapy (13). In our clinical case, the girl also has dysmorphic facial features but was not diagnosed with any additional genetic syndrome during exome testing. Sinescu et al. reported a case of a 3 year old child with WT and PKD on the left cystic kidney tumor and the right parenchymatous kidney tumor. Treatment involved right radical nephrectomy, partial left nephrectomy, chemotherapy, and radiotherapy. Nephrotic syndrome was diagnosed eight months after treatment, and the left kidney was removed. Hemodialysis was required before kidney transplantation, which was ultimately successful (14). In these three cases, nephrectomy was performed during the course of treatment. However, surgical treatment was not applied in our case. Based on the Umbrella protocol SIOP-RTSG 2016, after achieving a partial response to treatment, a second initial round of chemotherapy should be repeated. Chemotherapy proved to be very effective for our patient. Preoperative chemotherapy without biopsy is usually started when WT is suspected. Most patients with WT in Europe are treated with preoperative chemotherapy based on the SIOP guidelines. According to SIOP recommendations, tumor nephrectomy should be performed after preoperative chemotherapy for all patients after diagnosis. The stage of the disease is determined only after nephrectomy (15). In our clinical situation, a 2.0 × 1.9 cm mass in the background of a polycystic kidney raised doubts about its origin. Therefore, a biopsy of this mass was performed, and a blastemic type of Wilms tumor was found. After evaluating biopsy results and MRI changes, the diagnosis of left Wilms tumor with nephroblastomatosis was confirmed, and preoperative chemotherapy started according to the Umbrella protocol. After 12 weeks of preoperative chemotherapy, MRI tumor formations disappeared. De Jesus et al. described a 7 month old girl with bilateral nephromegaly diagnosed from birth. Ultrasound showed increasing nephromegaly with multiple heterogeneous hypoechoic nodules in both kidneys. Hyperplastic diffuse nephroblastomatosis (HDNBM) was suspected, and a percutaneous biopsy resulted in immature nephrogenic tissue. The girl was treated with chemotherapy based on the SIOP WT stage 1 protocol. After 6 chemotherapy cycles, all malignancy nodules in both kidneys regressed (10). In our case, since the patient had a chronic disease and tumor formations were not detected after preoperative chemotherapy, surgery was not performed. Treatment was given according to the Umbrella protocol AV2 with vincristine and actinomycin for 6 months, followed by the treatment of nephroblastomatosis for 1 year with vincristine and actinomycin every 28 days. The rarity of HDNBM, causing absent diagnostic consideration of the disease, absence of diagnostic suspicion, or primary diagnosis of cystic kidney disease, was chalenging to diagnose in our cases. Gargalionis et al. were the first to demonstrate in their study that polycystin-1 and polycystin-2 are associated with oncogenesis in colorectal cancer cells (11, 16). Another study by Jilg et al. reviewed 240 ADPKD patients who underwent kidney removal. The surgery was performed at a median age of 54 years. Histological examination of the removed kidneys was performed, and they found that 5% of the lesions were malignant (17). The most common malignant kidney tumor in reported PKD adult patients is kidney cell carcinoma (KCC). The frequency of KCC increased in dialyzed and transplanted patients. The occurrences of KCC stand at 3%–4%, a rate roughly 100 times greater than that found in the general population, with this risk escalating as the duration of dialysis extends (12, 18).

Kidney ultrasound is the primary method for diagnosing ADPKD in childhood. The disease can be identified in the fetus during gestation through ultrasound examination. Kidney cysts and diffusely enlarged kidneys with variable echogenicity may be diagnosed by prenatal ultrasonography (4, 19). In our clinical case report, polycystic kidney disease was suspected in the fetus during prenatal ultrasound and confirmed after birth. A better understanding of the prognosis of neonates with ADPKD is vital for prenatal genetic counseling (20). Approximately 3% of children with the diagnosed pathogenic variant of ADPKD experience an early onset and rapid progression of the disease. In the majority of patients, the kidneys massively enlarge, and the glomerular filtration rate significantly decreases in adulthood (5). ADPKD patients diagnosed at an early age are considered to have a high rate of mortality and severe complications (21). A study by Shamshirsaz et al. showed that in a cohort of 199 children, hematuria and proteinuria were associated with a larger kidney volume. Children with hypertension experienced faster kidney and cyst growth than their counterparts with normal blood pressure, which is indicative of a poor prognosis (20, 21). Our patient currently does not have proteinuria and hematuria but has arterial hypertension controlled by two antihypertensive drugs, which is a sign of a poor prognosis. Nonetheless, over 90% of ADPKD children diagnosed before 18 months maintain well-preserved kidney function into childhood (20). In our clinical case, WT was found incidentally. In children with polycystic kidney disease, only kidney ultrasound is routinely performed. In our case, MRI was performed to clarify the liver damage and the changes found in the kidneys, which were not observed by ultrasound. Treatment for Wilms' tumor is one of the notable achievements of pediatric oncology, with long-term survival rates exceeding 90% for cases with localized disease and more than 70% for cases with metastatic disease (22). Most often, Wilms' tumor affects one kidney, but in 6% of cases, both kidneys are affected. Bilateral WT are usually associated with pathogenic genetic variants and predisposing syndromes (23). Wetmore et al., in their study, sought to determine the risk of malignancy after kidney transplantation between patients with and without ADPKD. The occurrence of malignancies in recipients with polycystic kidney disease was 48% greater than anticipated in the general population. However, the median age of PKD recipients at transplantation was 51 years (24). Sun et al., in their article, describe that ADPKD cells and cancer cells share the same pathophysiological characteristics, such as increased abnormal cell proliferation and abnormal cell differentiation and polarity. Polycystin-1 and polycystin-2 play a role in regulating cell proliferation, differentiation, and the change of differentiation. Therefore, they recommend being alert for the development of malignancy processes in patients with ADPKD (25).

## Conclusion

Reports regarding whether a diagnosis of ADPKD is a risk factor for malignancy are conflicting. Children with ADPKD should be frequently monitored for complications such as hypertension, kidney insufficiency, and neoplasms like WT, even though it is rare. MRI is more advantageous than a kidney ultrasound for providing a diagnosis in the early stages of Wilms' tumor for patient with ADPKD. Monitoring is especially important when considering or preparing patients with ADPKD for kidney transplantation.

## Data Availability

The original contributions presented in the study are included in the article/Supplementary Material, further inquiries can be directed to the corresponding author.
